# Levels and Correlates of Numeracy Skills in Lebanese Adults with Diabetes: A Cross-Sectional Study

**DOI:** 10.3390/ijerph191710557

**Published:** 2022-08-24

**Authors:** Carmel Bouclaous, Luna Joe Azar, Nour Barmo, Ralph Daher, Jana Tabaja, Ghida El Hout, Lina Berika

**Affiliations:** Gilbert and Rose-Marie Chagoury School of Medicine, Lebanese American University, Byblos P.O. Box 36, Lebanon

**Keywords:** numeracy skills, diabetes mellitus, diabetes management, healthy lifestyles, non-communicable disease, prevention, Lebanon, DNT-15

## Abstract

Diabetes numeracy skills are required in the interpretation of food labels, insulin pump dosage, the interpretation of blood glucose meter data, and the determination of carbohydrate intake. This study assessed the levels and correlates of numeracy skills in Lebanese adults with diabetes to identify those most at risk of uncontrolled diabetes. In total, 299 adults with diabetes, mean age 47.4 ± 19.8 years, took the questionnaire. It consisted of self-developed items on sociodemographic and health-related factors, in addition to the Diabetes Numeracy Test-15 (DNT-15) and the Single Item Literacy Screener. Many participants (62%) scored < 10 on the DNT-15 indicating insufficient numeracy skills. DNT-15 scores were positively associated with literacy, exercise, healthy diet, perceived diabetes control, frequency of glycaemia measurement, ability to afford treatment, and ease of understanding information related to diabetes. Age, BMI, and complications were negatively correlated with DNT-15 score. Numeracy skills were higher in males, single individuals, and in people with type 1 diabetes, fewer complications, controlled HbA1c, higher income, higher education, a prior visit to a dietician, and ability to maintain personal care despite COVID-19. Interventions to strengthen numeracy skills would empower individuals with diabetes, lead to appropriate self-management behaviors, and prevent health complications in at-risk individuals.

## 1. Introduction

Diabetes is one of the fastest growing health challenges of the 21st century. Currently, 537 million (1 in 10) adults worldwide live with diabetes, and this number is expected to reach 784 million by 2045 [1]. Diabetes burden is not only attributed to prevalence but also to associated pathologies, namely neuropathy, retinopathy (possibly leading to blindness), nephropathy (possibly leading to kidney failure), skin problems such as gangrene, as well as premature morbidity and mortality due to heart and vascular diseases [2]. Diabetes is among the top 10 leading causes of death worldwide [3], responsible for 6.7 million deaths in 2021: that is a death every 5 s [1]. The global economic burden from diabetes and its complications is estimated to reach U.S. $2.1 trillion in 2030 [4]. For these reasons, the American Diabetes Association and the European Association for the Study of Diabetes recommend standardized diabetes education that focuses on dietary intervention, physical activity, glucose monitoring, insulin injection technique, insulin storage, recognition and treatment of hypoglycemia, and “sick day” rules [5].

In the Eastern Mediterranean Region, deaths from diabetes have more than doubled and represent the greatest percentage increase of all WHO regions [3]. Particularly in Lebanon, 12.9% of the adult population has diabetes, which puts the country in twelfth position for highest prevalence of diabetes in the region [6]. Complications were reported in 22% of cases, with retinopathy being most common [7]. This is mainly due to a deficiency in diabetes control caused by delays in visiting a specialized physician and performing standard testing such as HbA1c level, lack of adequate medical coverage, hypoglycemic episodes and diabetes-related complications [8].

Lebanese patients’ knowledge of diabetes and practice have been reported to be unsatisfactory with only 15.9% of patients knowing their medications’ side effects, 57% identifying the target HbA1c level, 78.7% recognizing the normal fasting blood glucose level, and 87.4% knowing the type of food to avoid for proper diabetes control [8]. Nonetheless, an educational intervention by a multi-disciplinary team with bi-weekly follow-up phone calls to type 2 diabetic patients helped achieve better glycemic control, better diabetes management self-efficacy, and self-care activities including proper diet, physical activity, weight control, medication adherence, foot care and stress management [9]. 

Poor health literacy has been shown to have an indirect effect on diabetes self-care and glycemic control [10], medication adherence [11], rates of complications especially retinopathies [12], concern beliefs about medication, and low self-efficacy in medication use [13]. More specifically, health numeracy is useful in the interpretation of glucose meter readings, and the calculation of carbohydrate intake and medication dosages; it also implies an ability to interpret risk, estimate time and measures, think logically and solve problems using math skills, and make appropriate health-related decisions [14]. Lower health numeracy has been associated with poor diabetes knowledge and self-management behaviors [15], poor diabetes self-efficacy [16], and suboptimal medication adherence [17], leading to worse glycemic control and more complications from diabetes [18]. However, poor numeracy skills are not detected easily in patients and often go unnoticed [19]. 

Lower diabetes numeracy skills in the United States have been associated with older age, non-white race, fewer years of education, lower reported income, lower literacy and general numeracy skills, lower perceived self-efficacy, and selected self-management behaviors as well as higher HbA1c [15]. In addition, these skills have been linked to lower confidence in using insulin pump features [20] and completing the multi-step calculations needed in the interpretation of food labels and the choice of adequate insulin dosage [21]. Countering this issue, health-literacy-sensitive diabetes management interventions were found to be effective in reducing HbA1c level [22]. These interventions entailed clear written and spoken communication between patient and health provider, empowerment of the patient to be more engaged in learning about the disease, and special considerations for language and culture. 

Literacy in the general Lebanese population has increased from 91% to 95% between 2009 and 2018 [23]. Despite that, 65.8% of the population have inadequate or problematic functional health literacy, which represents their ability to obtain health information through reading and writing [24]. Against this background, this cross-sectional study aims to assess numeracy skills in Lebanese individuals with diabetes to determine the extent to which these skills and their correlates influence diabetes self-care, and in doing so, identify individuals most at risk of complications and in need of added support and monitoring.

## 2. Materials and Methods

### 2.1. Study Participants

As per Cochran’s formula $\frac{Z^{2}.p\left(1-p\right)}{e^{2}}$ [25], with a confidence interval (CI) of 95%, a population proportion of Lebanese adults with diabetes of 0.129, and a margin of error of 0.04, the minimum sample size should be 270 participants. However, we adopted non-probabilistic sampling, and thus managed to recruit 299 participants. Eligible participants were Lebanese, 18 years of age or above, fluent in Arabic (the official language of Lebanon), and who had diabetes (type I, type II or gestational).

### 2.2. Data Collection

Ethical approval was obtained from the Institutional Review Board of the Lebanese American University (LAU.SOM.CB5.24/August/2020) and the Ethics Committees of the different hospitals. The questionnaire was created on google form and shared via a link to the electronic platform. Data collection was performed between November 2020 and December 2021. Participants were recruited by the research team from the endocrinology and family medicine outpatient clinics at the LAU Medical Center-Rizk Hospital (LAUMC-RH), Sacre Coeur Hospital, Notre Dame University Hospital, and the Diabetic Foot Center at LAUMC-RH. Others were recruited through the National Health Day (NHD), an event organized annually by the LAU Medical Students’ Association to provide free medical consultations in underserved communities. Participants completed the questionnaire while waiting for their appointment. All were invited to contribute to snowball sampling by sharing the survey link with relatives and friends who had diabetes. Participants were also recruited through DiaLeb, a non-governmental organization that promotes diabetes awareness, prevention and care, and an active member of the International Diabetes Federation. The research team also posted an invitation on open social support platforms on Facebook for individuals with diabetes in Lebanon and the link to the questionnaire was shared with those who showed interest. 

Persons with diabetes were given a short description of the study and its objectives. Screening questions asked the participant to confirm that they had diabetes, were Lebanese, were 18 years or older, and understood Arabic. Participants gained access to the survey after signing an informed consent. According to preference, the questionnaire was completed by the patients themselves or by the researcher entering the patient’s answers in a face-to-face interview. The latter was used with individuals who were illiterate, mainly recruited through the NHD. Patient’s confidentiality was maintained with the use of an anonymous survey. The subject was informed that participation was voluntary and that they could withdraw anytime without implications on their care. There were no incentives nor compensation for participating in the study.

### 2.3. The Questionnaire

The Arabic questionnaire consisted of the Diabetes Numeracy Test (DNT)-15 along with self-developed items for socio-demographics, diabetes-specific questions (type of diabetes, complications (more than one answer could be selected), HbA1c level (controlled at HbA1c < 7%, uncontrolled at HbA1c > 7%, and unknown when the participant does not recall or is not aware of their HbA1c level), ease of understanding information related to the disease, perceived control of diabetes, frequency of blood glucose level measurement, and COVID-19 pandemic-specific questions (reduced visits to healthcare professionals during the COVID-19 pandemic, decrease in overall diabetes self-care during the COVID-19 pandemic). There was also a question on desired lifestyle modifications including becoming more active, eating healthier food, improving medical compliance, and monitoring blood glucose level. 

The DNT-15 has 15 questions that assess diabetes-related numeracy skills pertaining to nutrition, exercise, glucose monitoring, oral medication, and insulin dosage. The items require mathematical skills such as addition, subtraction, multiplication, division, fractions, numerical hierarchy, and multistep calculations. The use of calculators is allowed. The DNT-15 has strong psychometric properties, covers all numeracy skills assessed by the original DNT, and has excellent internal reliability and construct validity [26]. The DNT-15 was validated in the Arabic language in Saudi Arabia [27]. In our study, minor edits were made to the Arabic translation to account for differences in dialect between Gulf and Lebanese Arabic. Items are scored as binary outcomes (correct or incorrect) with no partial credit given. A correct answer is scored 1 and an incorrect answer is given 0. Individual scores are added to obtain the total DNT-15 score, which may range between 0 and 15. A higher score indicates higher diabetes numeracy skills. 

The Arabic Single Item Literacy Screener (SILS) was used [28]. It enquires about one’s ability to read health information, and consists of a single question “How often do you ask someone for help to read the instructions and leaflets from a doctor or pharmacy?”. It is answered on a 5-point Likert scale ranging from ‘never’ to ‘always’. ‘Never’ and ‘rarely’ denote adequate reading skills, while ‘sometimes’, ‘often’, and ‘always’ indicate struggling with reading health resources [29].

### 2.4. Statistical Analysis

Analysis was carried out using the Statistical Package for the Social Sciences version 25. Continuous data were presented as mean values with standard deviation (SD) and categorical data as frequency counts and percentages. Spearman’s correlation was used to measure the degree of association between DNT-15 score (continuous variable) and other continuous or ordinal variables (age, BMI, SILS, exercise, healthy diet, perceived control of diabetes, frequency of blood glucose level measurement, ability to afford treatment, ease of understanding diabetes-related information). Two sample *t*-tests were performed to compare the means of two independent samples, namely the difference in DNT-15 mean score by sex, having income, visit to a dietician, follow-up visit to an endocrinologist, duration of diabetes, personal care and visits to the doctor affected during the COVID-19 pandemic, desire for lifestyle changes). A one-way Analysis of Variance (ANOVA) was used to analyze the difference between the means of more than two groups, in particular the difference between DNT-15 mean score and marital status, educational level, type of diabetes, and HbA1c level. Post hoc comparisons using the Tukey Honest Significance Difference test was used to make pairwise comparisons of these means. A *p* value of less than 0.05 was considered statistically significant. The DNT-15 has no cut-off point for sufficient versus insufficient diabetes numeracy skills; a median split was performed with values below the median constituting an insufficient numeracy skills category.

## 3. Results

The sample consisted of 299 participants with a mean age of 47.4 ± 19.8 years, and a slightly higher proportion of women (52%). 

### 3.1. Frequency Counts and Percentages

Table 1 provides the socio-demographic characteristics of the study participants. The median score on the DNT-15 scale was 10. Participants’ health-related characteristics are presented in Table 2. 

### 3.2. Spearman’s Correlation

The strength and the direction of association between the DNT-15 score and continuous or ordinal variables are reported in Table 3. Older age, higher BMI, and number of complications were negatively correlated with DNT-15 scores. However, SILS, ability to afford treatment, ease of understanding diabetes-related information, lifestyle (exercise and healthy diet), perceived control of diabetes, and frequency of blood glucose level measurement were positively correlated with DNT-15 scores.

### 3.3. Two Sample t-Tests 

#### 3.3.1. DNT-15 Scores and Sex 

There was a significant difference in DNT-15 scores between males (M = 9.85, SD = 3.12) and females (M = 8.72, SD = 3.49); t (296.56) = 2.9398, *p* = 0.003. These results suggest that male participants scored higher than female participants did.

#### 3.3.2. DNT-15 Scores and Having a Source of Income

There was a significant difference in DNT-15 scores between those who had income (M = 10.16, SD = 2.62) and those who did not (M = 8.51, SD = 3.69); t (288.69) = −4.4915, *p* < 0.001. These results suggest that participants who had an income scored higher than those who did not.

#### 3.3.3. DNT-15 Scores and Visit to a Dietician

There was a significant difference in DNT-15 scores between those who had visited a dietician (M = 10.07 SD = 3.12) and those who had not (M = 8.67, SD = 3.42); t (284.08) = −3.6936, *p* < 0.001. Participants who had visited a dietician scored higher than participants who had not.

#### 3.3.4. DNT-15 Scores and Follow-Up Visit to an Endocrinologist in the Past Three Months

There was no significant difference in DNT-15 scores between those who had a recent endocrinology follow-up visit (M = 9.65 SD = 3.47) and those who had not (M = 8.89, SD = 3.22); t (294.45) = −1.9656, *p* = 0.05. These results suggest that a follow-up visit to the endocrinologist did not affect participants’ score on the DNT-15.

#### 3.3.5. DNT-15 Scores and Decreased Personal Care during the COVID-19 Pandemic

There was a significant difference in DNT-15 scores between those who had experienced decreased personal care during the COVID-19 pandemic (M = 8.68 SD = 3.09) and those whose personal care was not affected (M = 9.74, SD = 3.51); t (295.09) = 2.774, *p* = 0.005. These results suggest that participants with lower DNT-15 scores had a decrease in their self-care during the COVID-19 pandemic. 

#### 3.3.6. DNT-15 Scores and Decreased Visits to the Doctor during the COVID-19 Pandemic

There was no significant difference in DNT-15 scores between those who experienced decreased visits to the doctor during the COVID-19 pandemic (M = 9.10 SD = 3.35) and those who did not (M = 9.65, SD = 3.37); t (297) = 1.285, *p*= 0.200. 

#### 3.3.7. DNT-15 and Desire to Make Lifestyle Changes 

There was no significant difference in DNT-15 scores between those who desired to make lifestyle changes (M = 9.24 SD = 3.25) and those who did not (M = 9.58, SD = 4.22); t (25.489) = −0.38421, *p* = 0.70.

#### 3.3.8. DNT-15 Scores and Diabetes Duration

There was no significant difference in DNT-15 scores between those who have had diabetes for less than five years (M = 9.01 SD = 3.45) and those who have had diabetes for five years and longer (M = 9.37, SD = 3.33); t (152.54) = −0.83473, *p* = 0.4.

### 3.4. One-Way ANOVA with Tukey HSD

#### 3.4.1. DNT-15 Scores and Marital Status

There was a significant effect of marital status on DNT-15 scores at *p* < 0.001 level for the three conditions (F (2, 296) = 10.17, *p* < 0.001). Post hoc comparisons using the Tukey HSD test indicated that the DNT-15 score for participants who were single was significantly higher than those who were married (*p* = 0.01, 95% C.I. = [−2.12, −0.18]). In addition, those who were single scored significantly higher than the divorced/widowed participants (*p* = 0.00, 95% C.I. = [−3.78, −1.12]). Married participants also scored significantly higher than the divorced/widowed participants (*p* = 0.04, 95% C.I. = [−2.60, −0.00]).

#### 3.4.2. DNT-15 Scores and Type of Diabetes 

We found a significant effect of diabetes type on DNT-15 scores at *p* < 0.05 level for the three conditions (F (2, 296) = 174, *p* = 0.00). Post hoc comparisons using the Tukey HSD test indicated that the DNT-15 score for type 1 diabetes was significantly higher than type 2 diabetes (*p* = 0.00, 95% C.I. = [−2.42, −0.49]). In addition, participants with type 1 diabetes scored significantly higher than those with gestational diabetes (*p* = 0.00, 95% C.I. = [−4.11, −0.47]). There was no significant difference in DNT-15 scores between participants with type 2 diabetes and those with gestational diabetes.

#### 3.4.3. DNT-15 Scores and HbA1c Levels 

A one-way between subjects ANOVA was conducted to compare the effect of HbA1c status on DNT-15 scores in participants with controlled, uncontrolled, or unknown HbA1c. There was a significant effect of HbA1c status on DNT-15 scores at *p* < 0.05 level for the three conditions (F (2, 296) = 8.514, *p* = 0.00). Post hoc comparisons using the Tukey HSD test indicated that the DNT-15 score for controlled HbA1c was significantly higher than that of uncontrolled HbA1c (*p* = 0.01, 95% C.I. = [0.18, 2.20]). In addition, the score for unknown HbA1c was significantly lower than that of controlled HbA1c (*p* = 0.00, 95% C.I. = [−3.19, −0.74]). There was no significant difference in DNT-15 scores between uncontrolled and unknown HbA1c (*p* = 0.34, 95% C.I. = [−2.07, 0.53]).

#### 3.4.4. DNT-15 Scores and Educational Level

There was a significant effect of educational attainment on DNT-15 scores at *p* < 0.05 level for the three conditions (F (3, 295) = 38.61, *p* < 0.001). Post hoc comparisons using the Tukey HSD test indicated that the DNT-15 score for participants with university education was significantly higher than for the illiterate (*p* = 0.00, 95% C.I. = [4.65, 8.66]), and also higher than for the primary educated (*p* = 0.00, 95% C.I. = [2.27, 4.50]) and secondary educated (*p* = 0.00, 95% C.I. = [0.29, 2.40]). In addition, DNT-15 score for participants with primary education was significantly higher than for illiterate participants (*p* = 0.00, 95% C.I. = [1.14, 5.39]). In addition, DNT-15 score for secondary education was significantly higher than for illiterate participants (*p* = 0.00, 95% C.I. = [3.21, 7.39]) and higher than the primary educated (*p* = 0.00, 95% C.I. = [0.77, 3.30]).

## 4. Discussion

This is the first study in Lebanon to assess numeracy skills among individuals with diabetes. Previous studies have focused on knowledge and practice of patients with diabetes [8], the quality of diabetes care [30] and the association between depression and diabetes care [31].

Close to 62% of our participants, with any type of diabetes, had insufficient numeracy skills. This finding was close to that reported (65%) among American patients with type 1 or type 2 diabetes [15] and much lower than another study (87.5%) focusing on patients with type 1 diabetes users of insulin pump therapy [20]. It is possible that patients with type 2 diabetes have lower engagement in healthy lifestyle changes, which reflects on their motivation to understand the nutritional value of food and apply their competencies during insulin management [32]. We also found that individuals with type 1 diabetes had higher numeracy skills than those with type 2 diabetes. Since the former are diagnosed relatively earlier, individuals with lower diabetes numeracy skills and longer duration of their diabetes may have had enough time to compensate for their numeracy deficit. Individuals with type 1 diabetes are usually diagnosed young and, therefore, taught about the disease at an earlier age and closely monitored by healthcare givers and parents throughout their childhood to decrease numeracy and self-care errors. They may also be more motivated to learn given that their life is dependent on appropriate dosing of insulin. Moreover, individuals with type 1 diabetes are more commonly treated with insulin, thus trained to have more advanced diabetes numeracy skills such as interpreting blood glucose meter data, administering medication dosages, and following specific nutritional recommendations [33]. Individuals with young onset type 2 diabetes tend to have a more severe disease course despite lifestyle modifications [34]. Compared to the abrupt onset of type 1 diabetes, type 2 has a rather insidious onset and could easily be missed. 

In Lebanon, some contextual factors such as a stressful lifestyle and post-war PTSD have been suggested as possible causes of sub-optimal self-care and medication adherence among individuals suffering of diabetes [35]. More recently, stress levels in the population have increased due to the 4 August 2020 explosion that caused more than 7000 injuries, 203 deaths and left 300,000 people homeless after 2750 tons of unattended ammonium nitrate detonated at the port [36]. This incident aggravated the crippling economic and financial crises, ranked among the top three most severe crises globally since the mid-nineteenth century [37]. 

Although our results showed slightly higher DNT-15 scores for individuals diagnosed more than five years ago in comparison to those diagnosed more recently, the difference was not significant. People who have had diabetes for a longer period may have adapted to their disease and become less resistant to the introduction of lifestyle modifications [38]. A case in point is that only 9% of our participants did not wish to introduce some form of lifestyle change involving physical activity, nutrition, medication adherence, blood glucose monitoring, and avoidance of complications. In addition, duration of diabetes has been associated with higher knowledge of diabetes, its treatments and complications in another context, namely a tertiary hospital in India [39].

Our findings show that higher education was associated with higher diabetes numeracy skills. This is expected, as diabetes-related mathematical and literacy skills may develop with increasing education. Patients with a lower level of education are at increased risk of type 2 diabetes [40] and show a steep decline in healthy habits such as physical activity [41] or consumption of fruits and vegetables [42]. Therefore, lower education and low diabetes numeracy skills are a poor indicator of diabetes self-care and management. This may be aggravated by a difficulty in understanding diabetes-related information, which was found to be correlated with DNT-15 scores. Concerning functional health literacy, the low number of participants who require help in reading written or printed health material (14%) may be due to their high level of education. There is sufficient evidence supporting a positive relationship between health literacy and self-care activities [43]. Health interventions and programmes have been shown to have a positive impact on health literacy in vulnerable groups such as the elderly, and evidence the efficacy on risk factors or actual disease [44]. In addition, a systematic review has shown that diabetes self-management significantly improved glycemic control in at least 80% of patients with type 2 diabetes in the Middle East [45]. Therefore, education on diabetes self-management is important and the collaboration between educators and physicians is necessary [46].

In Lebanon, education was put forward as a major means for rebuilding the country after the civil war of 1975–1990 [47]. Subsequently, post-civil war generations were more likely to be educated than older generations. Our study found that numeracy skills are negatively correlated with age. In fact, age is negatively correlated with diabetes knowledge in general [48]. As patients age, they experience greater decline in physical and functional status [49], possibly leading to lower numeracy skills. Moreover, patients with mild cognitive impairment were shown to have a decline in their health numeracy skills, i.e., difficulties in understanding numerical information pertaining to their health [50]. It is important to note that age as an individual factor may affect patients’ approach to self-care and their perception of health [51], which may also lead to depression and medication non-adherence among the elderly [52]. Moreover, cognitive impairment in older age would lead to worse self-care and pose challenges to elderly with diabetes, notably in diet and exercise [53]. Moreover, older people, unlike the young, need to perform more regular daily exercise to improve and sustain their insulin sensitivity, which puts them at a disadvantage [54].

Gender was significantly associated with diabetes numeracy skills in our study with males scoring higher than females. This result could be due to the discrepancy in the overall literacy rate between men and women in Lebanon because of higher enrollment of boys in primary education [55]. Women and girls could also suffer from limited and substandard services such as decreased access to healthcare centers and unaffordable consultations, particularly in rural areas. Of note, in a study in the United States, women were less likely to be treated with needed drugs and were less likely than men to reach the target HbA1c < 7.0% and target LDL-Cholesterol [56]. Consequently, diabetes is experienced differently by men and women, and targeting those differences is a much-needed step in future healthcare protocols [57]. 

In our study, patients with controlled HbA1c had significantly higher diabetes numeracy skills that those with uncontrolled levels. Intensive glycemic control to bring HbA1c percentage as close to normal as possible is a well-supported intervention [58]. Higher HbA1c levels have been associated with a greater cardiovascular and renal risk, particularly in patients with diabetes and known risk factors for these diseases. 

In addition, marital status of our participants was associated with numeracy skills, with single individuals scoring higher than the coupled who, in turn, scored higher than the divorced/widowed. This could be explained by the fact that widowed individuals, who are more likely to be elderly, could lack the social network of family and friends who could help in diabetes management and the adoption of a healthy lifestyle, and be more prone to elder self-neglect [59]. Moreover, sudden life changing events such as the loss of a spouse or a divorce could take a toll on one’s health [60]. Actually, married individuals have been reported to perform number comprehension and mental calculation tasks better than single/widowed individuals in a group of older adults (ages 64 to 94 years) [61].

Participants with an income and those who reported being able to afford treatment had higher numeracy skills than those who did not. Those results are directly related to diabetes self-management. This can be explained by a higher ability to afford health care visits and be better prepared to face the disease. A previous study in Lebanon has shown that health literacy was positively correlated with income, ability to pay for treatment, self-perceived health and educational level, and negatively correlated with age and chronic disease [24]. Similarly, household income and health insurance have been proposed as an explanation for medication non-adherence in patients with diabetes [52]. A systematic review evaluating gaps in diabetes guidelines in low- and middle-income countries (LMIC) versus high-income countries showed inadequate applicability, clarity and dissemination plan in LMIC [62]. Those gaps may be having repercussions at the individual level. 

Our results suggest that that there is no relationship between diabetes numeracy skills and follow-up visit to the endocrinologist in the past 3 months. However, participants who had ever visited a dietician, as well as those who had a healthy lifestyle (appropriate exercise, diet, and BMI) scored higher on diabetes numeracy skills. This result is expected since patients with diabetes experience difficulties in interpreting food labels as well as serving size, and their performance is highly correlated with their level of literacy and numeracy skills [63]. For a long time, health professionals and patients with diabetes have considered nutrition therapy as the most challenging aspect of diabetes care [64]. Patients with diabetes tend to lose only half the weight lost by non-diabetic patients undergoing the same intervention [65]. However, lifestyle intervention is proven to reduce the occurrence of severe retinopathy by 47% in patients with diabetes [66], and cardiovascular disease mortality by 17% [67]. This demonstrates the impact of follow-up visits on patient education and self-efficacy.

Self-efficacy, being one’s belief in their capacity to execute behaviors necessary to produce specific performance attainments [68], can fuel the self-care behavior process [69]. In fact, self-efficacy-focused education leads to a significant drop in HbA1c of 0.61%, approaching a clinically significant level [70]. In this regard, those who monitored their blood glucose level more frequently and those who perceived their diabetes as controlled had higher DNT-15 scores. 

Data from the Middle East and North Africa region highlight that a considerable proportion of patients with diabetes do not attend follow up appointments [71,72]. Due to the restrictive measures and the fear caused by the COVID-19 pandemic, 70% of our study participants reported fewer visits to the doctor than usual. Other barriers that may have prevented them from accessing healthcare services include the ongoing economic crisis, lack of transportation, and absence of psychosocial support [73,74]. Additionally, COVID-19 pandemic isolation guidelines may have further exacerbated the proportion of people that do not attend follow up visits.

New techniques to maintain follow up may be the way forward. For instance, telemedicine devices have led to significant reductions in blood pressure, body weight, and lipid profile among patients with type 2 diabetes who were previously lost to follow-up [75]. In addition, the use of technology in web-based applications and communication via transfer of data from patients’ glucometers, insulin pumps or sensors have proved to be effective [76]. Such innovative methods are needed at a time when the COVID-19 pandemic highly impacted diabetes care worldwide with a drop in access to insulin, decreased admission to hospital for patients in need of professional care, and many outpatient clinics shutting down [77]. 

We found that DNT-15 scores were negatively correlated with the number of health complications, which could be explained by the influence of numeracy skills on HbA1c levels. In fact, higher HbA1c levels in patients with diabetes can lead to more complications [58]. Optimal diabetes self-care practices decrease diabetes-related complications and dramatically impact disease progression [78]. In the Gulf countries, baseline diabetes care was found to be suboptimal, with lack of adequate steps to prevent or delay the development of diabetes complications [79], an observation that may apply to Lebanon. 

It is important to note that participants who intended to make lifestyle modifications had higher numeracy skills. The desire for self-management has been previously demonstrated to be positively correlated with DNT-15 [80]. Given the chronicity of diabetes, it is fundamental for individuals to be motivated to tackle the challenging aspects of the disease. Intervention strategies have focused on informing the patient, teaching a particular skill set, encouraging behavior change and upkeep, strengthening assistance from the environment, and promoting changes to the socio-cultural context in order to maximize the effect of these interventions [81].

### Limitations

There are a number of limitations to this study. It was performed during the COVID-19 pandemic at a time when confinement was mandatory and fear of contracting the disease may have affected the health-seeking behavior of individuals with diabetes. Although we included individuals with diabetes through the National Health Day (that targets underserved rural communities), and through DiaLeb NGO (that serves the whole of Lebanon), we cannot rule out oversampling from similar social groups. The three medical centers used as recruitment sites were all private health facilities. Moreover, snowball sampling might have led to sampling bias, as people would be more likely to refer to others with similar levels of education and possibly same numeracy skills, which decreases the diversity of the subjects. In addition, the inclusion of a diabetic foot center might have led to higher inclusion of participants with uncontrolled diabetes and major complications. Since the questionnaire was online, we may have failed to collect data from individuals with limited digital literacy or those who did not have access to the internet on their mobile devices. For participants who did not take the survey in one of the medical centers, the absence of face-to-face interaction with the research team may have compromised their comprehension and their need for clarifications. It was not possible to report the response rate because the online platform did not record the number of attempts versus the number of submitted questionnaires. Since participation was voluntary, individuals with worse numeracy skills may have been less motivated to complete the survey. Furthermore, had the data been available, it would have been interesting to check for potential difference in DNT-15 scores between those who completed the online survey on their own and those who were in the presence of the research team. The cross-sectional study design, although inexpensive, precludes from identifying causation between variables.

## 5. Conclusions

Close to 62% of individuals with diabetes had insufficient numeracy skills. Patients with low numeracy skills tended to be older, divorced/widowed, female, with type 2 diabetes, and lower education. In addition, they were observed to have more diabetes complications, decreased desire to make lifestyle modifications, uncontrolled HbA1c, an unhealthy diet, decreased physical activity, inability to afford treatment, low ability to understand diabetes-related information, low perceived control of the disease, and low frequency of blood glucose level measurement. Since numeracy skills greatly affect diabetes self-management, it is advisable to raise individuals’ self-efficacy or their belief in their capacity to perform these skills. This need became more apparent with the COVID-19 pandemic. Health professionals should pay close attention to individuals at risk of insufficient diabetes numeracy skills. This entails increasing health communication between provider and patient, helping individuals with diabetes feel in control of their disease, devising educational resources including videos, images, and one-on-one training to demonstrate the tasks in diabetes self-care that require numeracy skills, and providing continuous motivation and support. 

Interventions to strengthen numeracy skills would empower individuals with diabetes, lead to appropriate self-management behaviors, and prevent complications in at risk individuals.

## Figures and Tables

**Table 1:** Socio-demographic characteristics of study participants (N = 299).

Variables	N (%)
**Sex**	
*Males*	144 (48)
*Females*	155 (52)
**Marital status**	
*Single*	116 (39)
*Married*	136 (45)
*Divorced/Widowed*	47 (16)
**Educational level**	
*Illiterate (<3 years of schooling)*	15 (5)
*Primary (3–8 years of schooling)*	63 (21)
*Secondary (9–14 years of schooling)*	75 (25)
*University (3+ years of higher education)*	146 (49)
**Source of Income**	
*Yes*	163 (55)
*No*	136 (45)

**Table 2:** Lifestyle and health-related characteristics of study participants (N = 299).

Variables	N (%)
**BMI**	
*<18.5*	6 (2)
*18.5 to 24.9*	92 (31)
*25 to 29.9*	132 (44)
*≥30*	69 (23)
**Exercise**	
*None*	181 (60)
*1–2 times per week*	62 (21)
*≥3 times per week*	56 (19)
**Healthy diet**	
*None*	51 (17)
*1–2 days per week*	53 (18)
*≥3 days per week*	195 (65)
**Type of diabetes**	
*Type I*	101 (34)
*Type II*	176 (59)
*Gestational*	22 (7)
**Duration of diabetes**	
*<5 years*	86 (29)
*≥5 years*	213 (71)
Complications *	
*Ocular problems*	96 (32)
*Cardiovascular problems*	55 (18)
*Hypertension*	118 (39)
*Hypercholesterolemia/Hyperlipidemia*	86 (29)
*Obesity*	83 (28)
*Stroke*	15 (5)
*Neurological problems*	46 (15)
*Depression or anxiety*	64 (21)
*Asthma*	36 (12)
*Dental or gum problems*	58 (19)
*Lower limb problems*	63 (21)
*Hand problems*	42 (14)
*Dermatological problems*	54 (18)
*Gastrointestinal problems*	41 (14)
*Sexual problems*	23 (8)
*Thyroid problems*	32 (11)
*Nausea, vomiting, constipation, or diarrhea*	31 (10)
*Recurrent infections*	43 (14)
*Kidney problems*	39 (13)
*Recent surgery in the past 5 years*	35 (12)
*No problem*	58 (19)
**Ability to afford diabetes treatment**	
*Very difficult*	49 (16)
*Difficult*	65 (22)
*Average*	114 (38)
*Easy*	57 (19)
*Very easy*	14 (5)
**Ease of understanding information on diabetes**	
*Very difficult*	28 (9)
*Somewhat difficult*	65 (22)
*Somewhat easy*	99 (33)
*Very easy*	107 (36)
**Frequency of blood glucose level measurement**	
*Never*	74 (25)
*Not regularly*	62 (21)
*Once or twice weekly*	36 (12)
*≥Once daily*	127 (42)
Need for someone to read medical instructions (SILS) **	
*Always*	42 (14)
*Often*	33 (11)
*Sometimes*	64 (21)
*Rarely*	83 (28)
*Never*	77 (26)
**Decreased visits to healthcare professionals during COVID-19 pandemic**	
*No*	91 (30)
*Yes*	208 (70)
**Decreased personal care during COVID-19 pandemic**	
Yes	165 (55)
No	134 (45)
**Desired lifestyle changes**	
*No change*	26 (9)
*Become more active*	161 (54)
*Eat healthier*	177 (59)
*Comply to medication*	149 (50)
*Orderly monitor glucose*	149 (50)
*Decrease risk of complications*	144 (48)
*Find solutions on days when sick and glucose levels are out of range*	104 (35)
**Perceived control of diabetes**	
*No control*	42 (14)
*Some control*	96 (32)
*Completely under control*	161(54)
**Numeracy skills**	
*Insufficient (DNT-15 score < 10)*	185 (62)
*Sufficient (DNT-15 score ≥ 11)*	114 (38)

* Participant could select more than one answer; ** SILS: Single Item Literacy Screener.

**Table 3:** Correlation between DNT-15 scores and different variables.

	DNT-15 Score
Age	−0.22 **
Body Mass Index	−0.2 **
Number of complications	−0.27 **
Single Item Literacy Screener	0.41 **
Ability to afford treatment	0.34 **
Ease of understanding diabetes-related information	0.32 **
Exercise	0.21 **
Healthy diet	0.22 **
Perceived control of diabetes	0.23 **
Frequency of blood glucose level measurement	0.21 **

** is significant at *p* < 0.01; r = 0.1 to 0.29 weak correlation; r = 0.3 to 0.49 moderate correlation; a negative (−) value represents a negative relationship.

## Data Availability

The data presented in this study are available upon request from the corresponding author.
